# An improvement of carotid intima-media thickness and pulse wave velocity in renal transplant recipients

**DOI:** 10.1186/s12880-018-0263-7

**Published:** 2018-08-17

**Authors:** Zhaojun Li, Yan Qin, Lianfang Du, Xianghong Luo

**Affiliations:** 1 Department of Ultrasound, Shanghai General Hospital, Shanghai Jiaotong University School of Medicine, No.100 Hai Ning Road, Hongkou District, Shanghai, 200080 China; 2 Department of Urology, Shanghai General Hospital, Shanghai Jiaotong University School of Medicine, No.100 Hai Ning Road, Hongkou District, Shanghai, 200080 China; 3 Department of Echocardiography, Shanghai General Hospital, Shanghai Jiaotong University School of Medicine, No.100 Hai Ning Road, Hongkou District, Shanghai, 200080 China

**Keywords:** Renal transplantation, Carotid intima-media thickness, Arteriosclerosis, Pulse wave velocity

## Abstract

**Background:**

Renal transplantation can significantly improve the quality of life of patients with end stage renal disease (ESRD) who would otherwise require dialysis. Renal transplant (RT) recipients have higher risks of cardiovascular disease compared with general population. The carotid intima-media thickness (CIMT) and pulse wave velocity (PWV) have been used as the important predicting factor of vascular arteriosclerosis. Therefore, this study was to investigate the improvement of carotid intima-media thickness and pulse wave velocity in renal transplant recipients.

**Methods:**

Thirty-one patients with chronic kidney disease being treated with hemodialysis, 31 renal transplant recipients and 84 healthy control subjects were included to have the clinical evaluations and ultrasonography of bilateral carotid arteries. CIMT and PWV were independently measured by two ultrasonographers using the technique of ultrasonic radiofrequency tracking and correlated with arteriosclerosis risk factors. The progression of CIMT and PWV with age were analyzed by linear regression models, and the slopes of curves were compared using Z test.

**Results:**

Compared with the patients on hemodialysis, the CIMT was significantly lower in renal transplant recipients and healthy control. The PWV were higher in hemodialysis patients and renal transplant recipients than that of the subjects in control group. The progression is CIMT positively corelated with age and cumulative duration in renal transplant recipients and hemodialysis patients. In both hemodialysis patients and renal transplant recipients, age and cumulative time on dialysis were all positively correlated with the increase of PWV as well.

**Conclusions:**

Carotid intima-media thickness and pulse wave velocity is the predicting factors of developing arteriosclerosis, which were improved in renal transplant recipients.

**Electronic supplementary material:**

The online version of this article (10.1186/s12880-018-0263-7) contains supplementary material, which is available to authorized users.

## Background

Kidney transplantation is the standard treatment for patients with ESRD because it can significantly prolong the life of the patient, mainly by improving renal function to prevent progression of cardiovascular disease [1]. Advancement in immunosuppressant therapies and surgical techniques have significantly improved RT outcomes with surgical complications decreased from 30 to 10% and rejection rates sharply declined from 50% to less than 10% [2, 3]. Compared with dialysis patients, kidney transplant recipients have a 10-fold reduction in cardiac death [4]. However, although the transplanted kidney improves the renal and cardiac function, renal function is still lower than normal. Compared with the normal population, kidney transplant patients still have a 10-fold higher in cardiac death, and 50 times annual fatal or non-fatal cardiovascular events [1]. The progression of arteriosclerosis is closely related to a variety of risk factors and plays an important role in the development of cardiovascular and cerebrovascular events. [5]. Intima-media thickness (IMT) and pulse wave velocity (PWV) are regarded as the footprints of arteriosclerosis [6, 7]. Although studies reported that decline of IMT may occur in some patients who received renal transplant, few studies have investigated the change of PWV in renal transplant recipients. There we performed this study to assess carotid intima-media thickness (CIMT) and PWV in hemodialysis patients and renal transplant recipients, with the purpose of investigating the value of using carotid intima-media thickness (CIMT) and pulse wave velocity (PWV) as the predictive factor of vascular arteriosclerosis.

## Methods

### Subjects

This study involved 112 consecutive adult ESRD patients (age ≥ 18 years) from May 2015 to December 2016. Among the 112 patients, 50 peritoneal dialysis patients were excluded. All the patients who received the renal transplant had been treated with hemodialysis. Considering the effect of arteriovenous fistulas on arterial stiffness in hemodialysis patients, peritoneal dialysis patients without arteriovenous fistulas were excluded from the study. Thus, the final study subjects comprised of 62 ESRD patients, including 31 patients who received a single renal transplant (renal transplant recipients group) and 31 patients relied on hemodialysis (hemodialysis patients group). All the RT patients received the treatment of immunosuppression, including tacrolimus, steroids and mofetil mycophenolate. The clinical information of the subjects was extracted from our Hospital Renal Transplant database, which had been updated yearly since 1993. 84 sex- and age-matched healthy subjects (55 men and 29 women; age rang, 20–80 years) were recruited as controls. They have no history of chronic kidney disease, and the results for physical and laboratory examinations,including electrocardiography, echocardiography and blood tests of hepatic and renal function, are normal. The study was approved by the Institutional Review Board of Shanghai General Hospital (2014158). Written informed consents were provided by all participants.

#### Patient demographic characteristics

Demographic characteristics, including age, gender, comorbidities, actual treatment, smoking status, weight and height, were collected from the electronic database of our hospital. Cardiovascular risk factors, including systolic blood pressure (SBP), diastolic blood pressure (DBP), fasting blood glucose (FBG), hemoglobin A1c (HbA1c), total cholesterol (TC), triglyceride (TG), low-density lipoprotein cholesterol (LDL-C) and high-density lipoprotein cholesterol (HDL-C), were recorded for all subjects. Diabetes was diagnosed as fasting plasma glucose levels ≥126 mg/Dl at the time of entry in this study, or if the individual who was undergoing treatment with a hypoglycemic agent or any long-acting insulin, was diagnosed diabetic patients. Hypertension was defined as systolic pressure ≥ 140 mmHg and/or diastolic pressure ≥ 90 mmHg, or continuous on the antihypertensive medications. Subjects were classified as smokers if they had smoked at least one 20-cigarette pack per day in the year before the study. Body mass index (BMI) was calculated as weight (kg) divided by the square of height (m2). Serum calcium (Ca) and phosphorus (P) were tested. Parathormone (PTH) concentrations were measured with the ELISA method (Diagnostic System Laboratories, Webster, TX, USA).

### Carotid ultrasonography

**Fig. 1:**
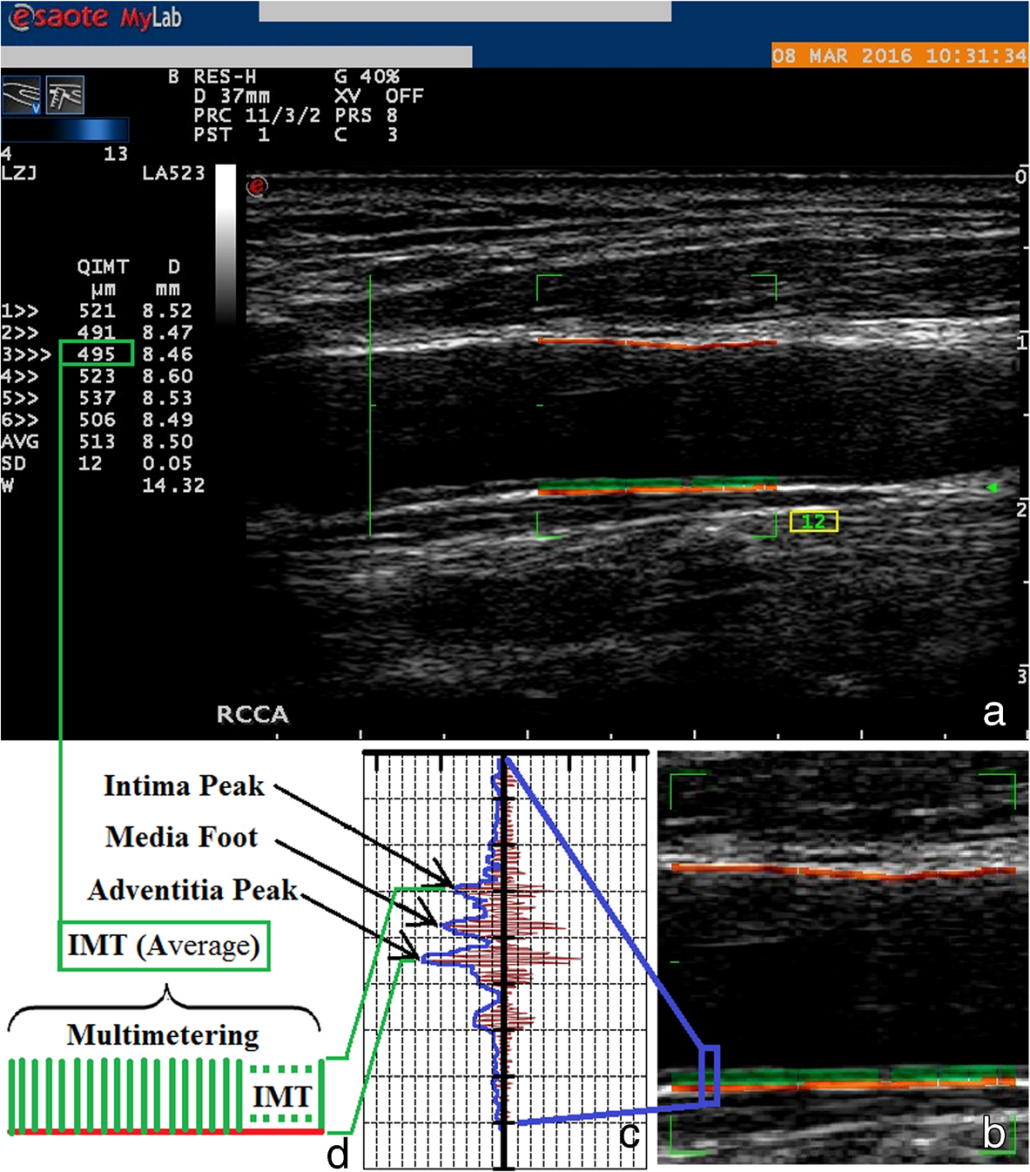
The Measurement of CIMT and PWV using ultrasonic radiofrequency tracking technique. **a** Analysis of CIMT and PWV using the ultrasound system equipped with the assoated software. **b** Magnification of the region of interest. **c** Ultrasonic RF signal diagram for single IMT point. **d** Assessment of multipoint IMT on segmental carotid artery

All ultrasonographic measurements were performed by two ultasonographers who were blinded to the clinical data (ZJ Li and LF Du, with 16 and 30 years of experience in ultrasound diagnosis, respectively), as described previously [8]. Ultrasound examinations were conducted with the Mylab Twice ultrasonographic diagnostic system (ESAOTE Medical Systems, Genova, Italy), equipped with a 4–13 MHz linear array transducer and intima-media thickness (QIMT) and arterial stiffness (QAS) quantity software (Fig. 1). After the subject was placed in supine position, the common carotid artery (CCA) was shown in a longitudinal view. The imaging acquisition was focused on 1.5-cm segment proximal to the dilatation of the bifurcation. The anterior and posterior walls were depicted clearly. Following the entry of blood pressure while acquiring the imaging date, with the initiation of QIMT and QAS functions, the RF signals tracking the vascular walls for at least six cardiac cycles. The local carotid systolic pressure (Loc-Psys), diastolic pressure (Loc-Pdia), CIMT and PWV were automatically recorded.

Specifically, PWV was calculated from the following equation:

$$ \mathrm{PWV}=\frac{1}{\sqrt{\rho \cdot DC}}=\sqrt{\frac{D^2\cdot \Delta P}{\rho \cdot \left(2\cdot D\cdot \varDelta D+\varDelta {D}^2\right)}} $$

[9].

Where, *D* = diastolic diameter, Δ*D* = change of diameter in systole and DC = distensibility coefficient, Δ*P* = local pulse pressure, *ρ* = blood density. PWV is a functional parameter directly affected by arterial wall stiffness. In addition to these hemodynamic parameters, the peak systolic velocity (*V*_max_), end diastolic velocity (*V*_min_), mean flow velocity (*V*_mean_), velocity time integral (VTI), artery S/D ratio, resistance index (RI) and pulsatility index (PI) of the CCA were also recorded by using vascular ultrasound measurement. RI and S/D ratio were calculated using the following formulas:

$$ \mathrm{RI}=\left({V}_{\mathrm{max}}-{V}_{\mathrm{min}}\right)/{V}_{\mathrm{max}} $$

$$ \mathrm{PI}=\left({V}_{\mathrm{max}}-{V}_{\mathrm{min}}\right)/{V}_{\mathrm{mean}} $$

$$ \mathrm{S}/\mathrm{D}={V}_{\mathrm{max}}/{V}_{\mathrm{mean}} $$

### Statistical analysis

Continuous data were expressed as mean ± SD. The one-way analysis of variance analysis and least significant difference method were used to compare the differences between the groups. The Chi-square test was used for the comparisons of categorical variables between the groups. Repeatability evaluation between the two observers using linear correlation analysis and Bland-Altman Plots. Subsequently, variables related to the carotid morphological were correlation with stiffness parameters by using the stepwise multiple linear regression models. The slops of the CIMT-to-Age and PWV-to-age were determined using linear regression models. *Z* test was used for the comparison of slopes between groups. The statistical analyses were performed using SPSS 13.0 (SPSS, Chicago, IL) and the statistical significance set at *P* < 0.05.

## Results

### Characteristics of the subjects

Basic clinical characteristics and laboratory results were described in Table 1. transplant recipients had higher DBP than hemodialysis patients, while no significant difference of SBP between hemodialysis patients and RT patients exist. Level of serum phosphorus was higher in hemodialysis patients, comparing with the transplant recipients and control groups. There was no significant difference of cumulative time on dialysis between the hemodialysis patients group and hemodialysis patients. No difference of age, BMI, TG, TC, LDL-C, HDL-C, FBG, and HbA1c exist among the three groups.

**Table 1:** Clinical characteristics of three groups

Variables	Controls (*n* = 84)	Hemodialysis(*n* = 31)	RT (n = 31)	^a^*P* Values
Age (years)	58.1 ± 19.9	59.3 ± 17.9	57.9 ± 14.3	0.141
Male, n (%)	55(65)	20(65)	22(71)	0.317
Body-mass index (kg/ m^2^)	26.2 ± 4.5	26.0 ± 5.5	24.2 ± 3.5	0.083
Time in predialytic ERSD (mo)	–	70(1–148)	73(1–216)	0.351
Cumulative time on dialysis (mo)	–	24(1–94)	26(1–104)	0.561
Hypertension, n (%)	11(12)	28(90)^†^	25(80)^†^	0.039
Diabetes mellitus, n (%)	11(13)	9(28)^†^	8(27)^†^	0.025
Dyslipidemia, n (%)	4 (5)	2(6)	2(6)	0.676
Smokers, n	4	3	1	0.07
Systolic blood pressure (mmHg)	119.3 ± 15.8	146.9 ± 21.3^†^	145.8 ± 13.5^†^	<0.001
Diastolic blood pressure (mmHg)	77.1 ± 8.3	86.9 ± 13.5^†^	94.3 ± 8.6^†‡^	<0.001
Glycosylated hemoglobin A1c (%)	4.6 ± 0.7	4.7 ± 0.7	4.9 ± 0.6	0.156
Total cholesterol (m mol / L)	6.0 ± 1.2	4.9 ± 1.2	4.5 ± 0.8	0.322
Triglycerides (m mol / L)	1.4 ± 0.7	1.9 ± 0.9	1.4 ± 0.3	0.345
Low-density lipoprotein (m mol / L)	3.1 ± 0.9	2.7 ± 0.9	2.3 ± 0.6	0.224
High-density lipoprotein (m mol / L)	1.2 ± 0.3	1.3 ± 0.3	1.4 ± 0.3	0.432
Fasting glucose (m mol / L)	5.3 ± 1.1	4.6 ± 0.6	4.9 ± 0.7	0.245
Ca (m mol / L)	2.39 ± 0.10	2.40 ± 0.14	2.33 ± 0.19	0.422
P (m mol / L)	1.33 ± 0.12	1.79 ± 0.24†	1.39 ± 0.56^#^	0.003
PTH (pg/ml)	35 ± 26	27 ± 20†	39 ± 22^#^	0.050
ACEI use, n (%)	5(6)	17(56)^†^	20(64)^†^	0.038
Calcium channel antagonist, n (%)	4(5)	16(50)^†^	10(32)^†^	0.032
Diuretics, n (%)	4(5)	12(40)^†^	6(18)^†#^	0.042
Beta-blockers, n (%)	4(5)	12(40)^†^	8(25)^†#^	0.036
Statin use, n (%)	15(18)	11(35)^†^	10(33)^†^	0.046

^a^In three groups, Chi-squared test or ANOVA test was used to compare the distribution of age, gender, BMI, Time in predialytic ERSD, Cumulative time on dialysis, hypertension, hyperlipidemia, diabetes mellitus, Dyslipidemia, smoking, SBP, DBP, HbA1c, TC, TG, LDL, HDL, FBG, Ca, P, and PHT

^*^P<0.05, ^†^*P* < 0.01 compared with the control group; ^#^*P*<0.05, ^‡^*P* < 0.01 compared with the hemodialysis group. Data presented as mean (SD) or n (%)

### Comparison on repeatability

The median CIMT measured by the two observers were 505 μm (IQR, 397–599 μm) and 526 μm (IQR, 421–594 μm). The median PWV were 7.2 m/s (IQR, 5.4–8.6 m/s) and 7.1 m/s (IQR, 5.4–8.7 m/s), respectively. The ICC for the CIMT and PWV measured by the two observers was 0.93 for PWV (95% confidence interval [CI]: 0.91, 0.94) and 0.95 for CIMT (95% CI: 0.94, 0.98). Combining Bland-Altman and linear correlation analysis in intergroup, the results were further confirmed the measurement of CIMT and PWV was a consistent trend. (Additional file 1: Figures S1-S4).

### Comparison of carotid structure, function and hemodynamics

The morphologic, functional and hemodynamic analyses result of carotid arteries were provided in Table 2. Compared with the hemodialysis patients group, the CIMT was significantly lower in both the renal transplant group and control group. No significant difference of CIMT was found between the renal transplant recipients and the control group. PWV of both the hemodialysis patients and renal transplant recipients were all higher than that in the control group. No significant difference of PWV was found between the renal transplant group and the hemodialysis patients. Compared with the control group, the *V*_max_, PI and S/D were significantly lower in the renal transplant recipients. The three groups showed no significant difference in VTI, *V*_min_, *V*_mean_, and RI.

**Table 2:** Comparision of sonographic carotid artery measures in three groups

Variables	Controls (n = 84)	Hemodialysis (n = 31)	RT (n = 31)	^a^*P* Values
Carotid intima-media thickness (μm)	529.7 ± 131.8	561.9 ± 147.7^*^	480.5 ± 90.3^#^	0.045
Pulse wave velocity (m/s)	6.68 ± 2.25	7.87 ± 2.25^*^	8.05 ± 2.17^†^	0.004
Velocity time integral (m)	0.2 ± 0.1	0.2 ± 0.1	0.2 ± 0.1	0.250
Peak systolic velocity (cm/s)	64.4 ± 19.9	56.6 ± 20.3	49.9 ± 14.5^†^	0.002
End diastolic velocity (cm/s)	16.27 ± 6.5	17.5 ± 10.5	15.9 ± 7.3	0.702
Mean flow velocity (cm/s)	26.9 ± 7.4	27.5 ± 10.1	24.8 ± 7.7	0.388
Pulsatility index	1.8 ± 0.6	1.6 ± 0.7^*^	1.4 ± 0.4^†^	0.003
Resistance index	0.7 ± 0.2	0.7 ± 0.2	0.7 ± 0.2	0.197
S/D ratio	4.3 ± 1.2	4.1 ± 2.1	3.4 ± 0.9^†^	0.017

^a^In three groups, Chi-squared test or ANOVA test was used to compare the distribution of CIMT, PWV, VTI, PSV, EDV, MFV, PI, RI and S/D ratio

^*^P<0.05, ^†^*P* < 0.01 compared with the control group; ^#^*P*<0.05, ^‡^*P* < 0.01 compared with the hemodialysis grou*p*

### Impact of risk factors on CIMT and PWV

CIMT was positively correlated with Age and SBP in both the control group and renal transplant recipients, however negatively correlated with age in hemodialysis patients. In the hemodialysis patients and renal transplant recipients, CIMT was positively correlated with cumulative time on dialysis. No significant correlation between CIMT and SBP was found in the hemodialysis patients (Table 3).

**Table 3:** Multiple linear regression analysis of carotid intima-media thickness, carotid diameter versus cardiovascular risk factors

Variables	Carotid intima-media thickness			Pulse wave velocity		
	Control	HD	RT	Control	HD	RT
	β^a^(β^b^)	β^a^(β^b^)	β^a^(β^b^)	β^a^(β^b^)	β^a^(β^b^)	β^a^(β^b^)
Age	6.91 (0.84)^‡^	−1.40(− 0.19)^†^	2.38 (0.30)^†^	0.07(0.64) ^‡^	0.07(0.57) ^‡^	0.08 (0.50) ^†^
CTD	/	0.39(0.24)^‡^	1.81 (0.23)^†^	/	0.53(0.17)^†^	0.58 (0.21)^†^
PI	−101.42 (−0.37)	−197.74(− 1.14)	162.70(0.78)	− 0.43(− 0.10)	−1.26(− 0.38)	2.12 (0.43)
RI	662.43(0.62)	246.21 (0.49)	− 469.76 (− 0.78)	−0.04(− 0.01)	0.60(0.06)	−9.10 (− 0.67)
EDV	10.84(0.53)^†^	−2.96 (1.14)	− 15.61 (− 0.74)	−0.01(− 0.03)	0.01(0.04)	−0.08 (− 0.23)
MFV	−20.47 (− 0.96)^†^	−9.67 (− 0.70)	14.60 (0.74)	−0.02(− 0.05)	0.01(0.01)	0.04 (0.13)
SBP	−32.38(−0.29)^†^	51.59 (0.90)	− 37.73(− 0.46)^†^	0.04(0.26)^‡^	0.03(0.29)	0.04 (0.27)

^a^:Unstandardized Coefficients;^b^:Standardized Coefficients;CTD: Cumulative time on dialysis. †P < 0.05,‡P < 0.01

In the control group, PWV were positively correlated with age and SBP (*r* = 0.07 and 0.04, all *P*<0.05), respectively. In both hemodialysis patients and renal transplant recipients, PWV were all positively correlated with age and cumulative time on dialysis (In HD group, *r* = 0.07, 0.53 and In RT group, *r* = 0.08, 0.58, all *P*<0.05), respectively (Table 3).

### Tendency of CIMT and PWV with age

**Fig. 2:**
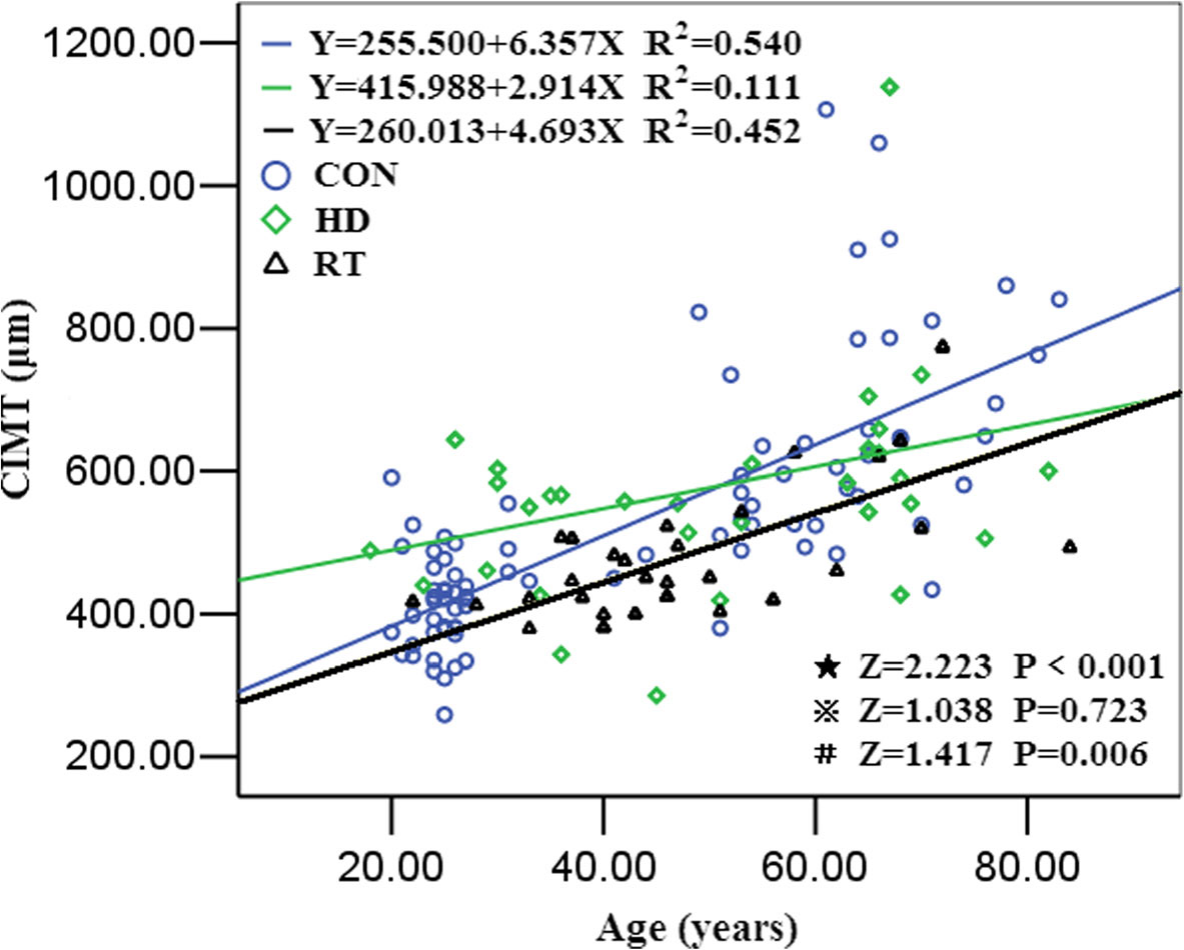
The linear regression curve of carotid intima-media thickness with age in the three groups. ⋆control group VS. hemodialysis group, ⋇hemodialysis group VS. RT group, #control group VS. RT group

**Fig. 3:**
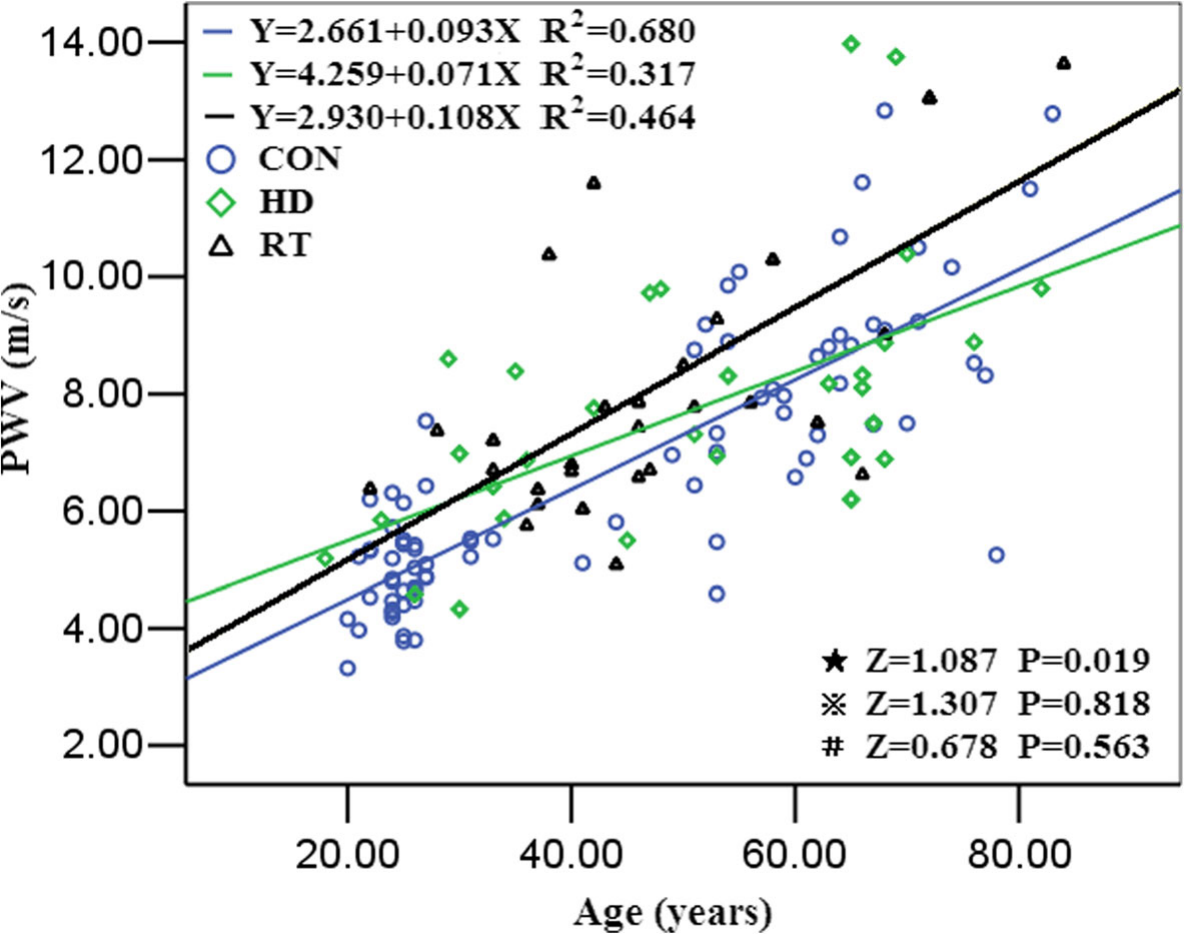
The linear regression curve of pulse wave velocity with age in the three groups. ⋆Control group VS. HD group, ⋇HD group VS. RT group, #Control group VS RT group

The age-CIMT curves of the 3 groups showed that the slopes of curves were 6.357, 4.693, and 2.914 in the control group, the renal transplant recipient group, and the hemodialysis patients group, respectively. The pairwise comparisons showed that the slopes of the age-CIMT curve of the control group was higher than the renal transplant recipient group (Z = 1.417, *P* = 0.006) and the hemodialysis patient group (Z = 2.223, *P*<0.001), respectively. No significant difference was found between the renal transplant recipient group and the hemodialysis patient group (Z = 1.038, *P* = 0.723) (Fig. 2). PWV trended to increase with age and the positive correlation between PWV and age were showed in the 3 groups. Compared with the control group, the age-PWV slope was significantly lower in the hemodialysis patients (Z = 1.087,*P* = 0.019. No difference was found between the slope of the age-PWV curve in the renal transplant recipient and that in both control and hemodialysis patients (Z = 0.6787, and *P* = 0.563) and between the renal transplant recipients and hemodialysis patients (Z = 1.307,*P* = 0.818) (Fig. 3).

## Discussion

It remains in debate whether renal transplantation could retard the progression of atherosclerosis in patients with end-stage renal disease. In this study, the technique of ultrasonic radiofrequency tracking was used to quantify the CIMT and PWV, with the recording of up to 50,000 images per second and acquisition of 30–50 samples of one 1–1.5-cm segment of carotid artery, which allows the precise measurement of CIMT and PWV at the micron level and ensures the great repeatability. Our study shows that CIMT and PWV values were lower in age-matched renal transplant recipients than in hemodialysis patients.

A few studies reported the measurement of CIMT using conventional ultrasound in two dimensions and showed that the CIMT increased after renal transplant [10]. Nafar et al. followed up 26 renal transplant recipients and found that the CIMT of carotid arteries increased after renal transplant. This trend of CIMT increase continued during the 2th, 4th, and 6th month ([0.85 ± 0.22], [0.87 ± 0.23], and [0.88 ± 0.24] mm, respectively) post renal transplant [11]. Mitsnefes et al. found that children had higher CIMT after kidney transplant than pre-transplantation ([0.42 ± 0.07] mm vs. [0.38 ± 0.06] mm), and the CIMT value was closely related to SBP [12]. However, in our study, the CIMT was smaller in renal transplant recipients than in hemodialysis patients, and there was no significant difference of CIMT between the renal transplant recipients and the control group. Danielson et al. published their study on the patients with type 1 diabetes after pancreatic islet transplantation [13], showing a significant decrease in CIMT after islet transplantation in individuals with type 1 diabetes. 12 months after transplant, CIMT decreased by 0.062 mm (0.801pre-0.739post = − 0.062 mm). At the time of 50 months after transplant, CIMT significantly decreased by 0.026 mm (0.801pre-0.775post = − 0.026 mm). Their study showed that that the decrease of CIMT was associated with the decrease of HbA1c, suggesting CIMT could improve after the major risk factors of artery stiffness were removed. Our study showed the similar phenomena that renal transplantation may offer the benefit of removing the risk factors of atherosclerosis in the patients with ESRD, which was manifested as the decrease of CIMT in renal transplant recipients. Our study found that CIMT was positively correlated with the ages of patients.

Age is an important risk factor for the development of arteriosclerosis in the elastic arteries (e.g. aorta and carotid artery) [14]. Age-related arterial remodeling was significantly accelerated by hemodialysis therapy [15]. In this study, the slopes of CIMT-to-age were lower in renal transplant recipients than that in hemodialysis patients. Domingo et al. reported the similar results that the CIMT increased in the trend of age increase [16]. This result suggested that the primary risk factors (e.g. chronic renal insufficiency, arteriovenous fistula, etc.) were removed, and arterial remodeling was improved after renal transplantation.

PWV is another major parameter being used to assess the development of arteriosclerosis [17–19]. In this study, PWV in the hemodialysis patients was higher than that in the control group, but no significant increase was found compare with that of the transplant recipients. A few studies presented the similar results. The study by Birdwell et al. showed that no PWV increase (median: baseline 9.25 to 12-month 8.97 m/s) was detected in the group of 66 new renal transplant recipients within a follow-up period of 12 months [20]. In Bachelet-Rousseau’s et al. study, in 88 patients, including 39 transplanted patients and 49 transplantation-pending patients, no significant difference of PWV between transplanted patients and transplantation-pending patients was observed, with the PWV of 9.2 (7.9–11.9) m/s and 9.8 (7.7–12.1) m/s, respectively [21], which can be explained that the restoration of renal function after transplantation had a positive effect on slowing the progression of arterial stiffness. Arterial stiffness was associated with many risk factors. Age was recognized to be an independent risk factor of arterial elasticity. Our study showed that the higher regression coefficients between age and PWV were shown in hemodialysis patients than transplant recipients (β = 0.071 and 0.108, respectively), which indicates that the progression of vascular stiffness was improved in the transplant recipients. After renal transplantation, the cardiovascular risk factors of kidney disease, including disorders of calcium and phosphorus metabolism and the hemodynamics factors are improved, which bring the improvement on the development of arterial stiffness [22]. In addition, immunosuppressant drugs can reduce vascular inflammatory reaction and improve the progression of arteriosclerosis. Therefore, the effect of age background on RT patients was increased and the weights of other risk factors was decreased,with a different set of that in hemodialysis patients. Covic et al. showed that PWV was decreased in transplant recipients ([6.59 ± 1.62] m/s), while it was increased when patients ([7.19 ± 1.88] m/s) were receiving dialysis treatment [23].

There were some limitations in this study. This is an observational single-center study with relatively small sample size. A longitudinal study with large sample size are needed to evaluate the morphologic changes of carotid arteries and their association with cardiovascular factors (e.g., anemia, transplant time, etc.).

## Conclusions

In conclusion, our study demonstrated that the significantly lower PWV and CIMT in transplant recipients than that in the patients with hemodialysis, which indicate that PWV and CIMT may be used as the predictive factor for the improvement of atherosclerosis progression.

## Additional file

Additional file 1: **Figure S1.** Repeatability was analyzed by Bland-Altman Plots in intergroup. Bland-Altman analysis showed a consistent trend in the difference value and the mean value of CIMT by repeated measurement. **Figure S2.** Repeatability was analyzed by linear correlation analysis in intergroup. The results showed that intergroup comparison had a high degree of consistency. **Figure S3.** Repeatability was analyzed by Bland-Altman Plots in intergroup. Bland-Altman analysis showed a consistent trend in the difference value and the mean value of PWV by repeated measurement. **Figure S4.** Repeatability was analyzed by linear correlation analysis in intergroup. The results showed that intergroup comparison had a high degree of consistency. (DOCX 104 kb)

## Abbreviations

CI: Confidence interval
CIMT: Carotid intima-media thickness
ESRD: End-stage renal disease
FBG: Fasting blood glucose
HbA1c: Hemoglobin A1c
HD: Hemodialysis
HDL-C: High-density lipoprotein cholesterol
ICC: Intraclass correlation coefficient
IQR: Interquartile range
LDL-C: Low-density lipoprotein cholesterol
PWV: Pulse wave velocity
RT: Renal transplant
SD: Standard deviation
TC: Total cholesterol
TG: Triglyceride
