# Supplementary material for: An improvement of carotid intima-media thickness and pulse wave velocity in renal transplant recipients
Source: BMC Med Imaging. 2018 Aug 17;18:23. doi: 10.1186/s12880-018-0263-7 (PMC6098595; doi:10.1186/s12880-018-0263-7)
Supplement: Supplementary file 1 — Figure S1. Repeatability was analyzed by Bland-Altman Plots in intergroup. Bland-Altman analysis showed a consistent trend in the difference value and the mean value of CIMT by repeated measurement. Figure S2. Repeatability was analyzed by linear correlation analysis in intergroup. The results showed that intergroup comparison had a high degree of consistency. Figure S3. Repeatability was analyzed by Bland-Altman Plots in intergroup. Bland-Altman analysis showed a consistent trend in the difference value and the mean value of PWV by repeated measurement. Figure S4. Repeatability was analyzed by linear correlation analysis in intergroup. The results showed that intergroup comparison had a high degree of consistency. (DOCX 104 kb) [file 12880_2018_263_MOESM1_ESM.docx]

**Supplementary Figure Legends**


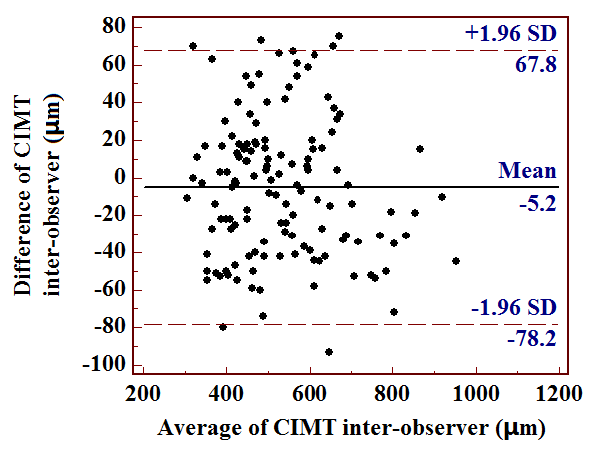


**Figure S1.** Repeatability was analyzed by Bland-Altman Plots in intergroup. Bland-Altman analysis showed a consistent trend in the difference value and the mean value of CIMT by repeated measurement.


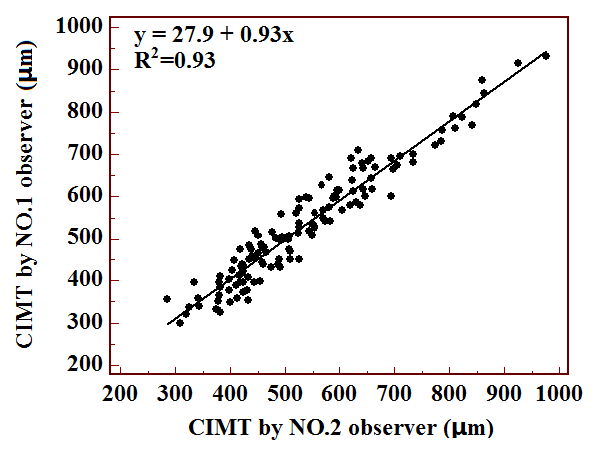


**Figure S2.** Repeatability was analyzed by linear correlation analysis in intergroup. The results showed that intergroup comparison had a high degree of consistency.


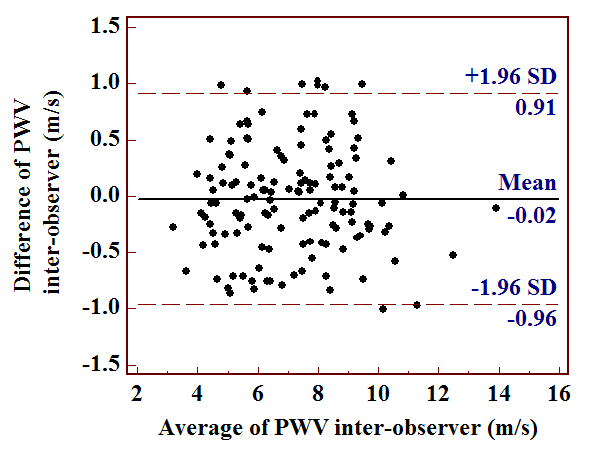


**Figure S3.** Repeatability was analyzed by Bland-Altman Plots in intergroup. Bland-Altman analysis showed a consistent trend in the difference value and the mean value of PWV by repeated measurement.


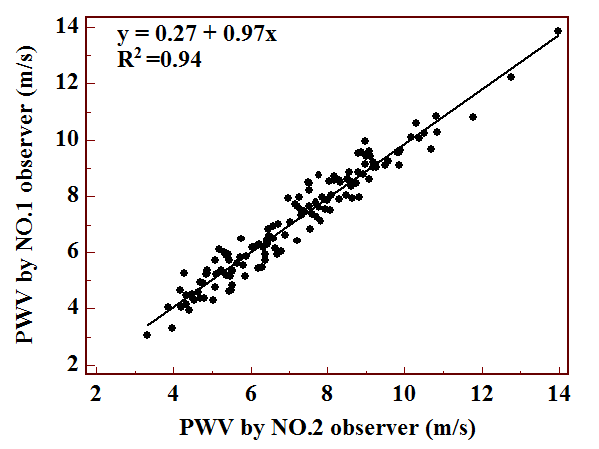


**Figure S4.** Repeatability was analyzed by linear correlation analysis in intergroup. The results showed that intergroup comparison had a high degree of consistency.
